# Comparison of uterine and tubal pathology identified by transvaginal sonography, hysterosalpingography, and hysteroscopy in female patients with infertility

**DOI:** 10.1186/s40738-015-0012-3

**Published:** 2015-12-23

**Authors:** Catherine H. Phillips, Carol B. Benson, Elizabeth S. Ginsburg, Mary C. Frates

**Affiliations:** 1 Department of Radiology, Brigham and Women’s Hospital, Harvard Medical School, 75 Francis Street, Boston, MA 02115 USA; 2 Department of Infertility and Reproductive Surgery, Obstetrics and Gynecology, Brigham and Women’s Hospital, Harvard Medical School, 75 Francis Street, Boston, MA 02115 USA

**Keywords:** Infertility, Transvaginal sonography, Hysterosalpingography

## Abstract

**Background:**

The causes of female infertility are multifactorial and necessitate comprehensive evaluation including physical examination, hormonal testing, and imaging. Given the associated psychological and financial stress that imaging can cause, infertility patients benefit from a structured and streamlined evaluation. The goal of such a work up is to evaluate the uterus, endometrium, and fallopian tubes for anomalies or abnormalities potentially preventing normal conception. To date, the standard method for assessing these structures typically involves some combination of transvaginal sonography (TVS), hysterosalpingography (HSG), and hysteroscopy (HSC). The goal of this review is to compare the diagnostic accuracy of TVS, HSG, and HSC for diagnosing abnormalities in infertility patients to determine if all studies are necessary for pre-treatment evaluation.

**Results:**

We identified infertility patients prior to initiation of assisted reproductive technology who had baseline TVS, HSG, and HSC within 180 days of each other. From medical record review, we compared frequencies of each finding between modalities. Of the 1274 patients who received a baseline TVS over 2 years, 327 had TVS and HSG within 180 days and 55 patients had TVS, HSG and HSC. Of the 327, TVS detected fibroids more often than HSG (74 vs. 5, *p* < .0001), and adenomyosis more often than HSG (7 vs. 2, *p* = .02). HSG detected tubal obstruction more often than TVS (56 vs. 8, *p* = .002). Four (1.2 %) patients had endometrial polyps on both HSG and TVS.

In the 55 patients with HSG, TVS, and HSC, HSC identified endometrial polyps more often than TVS (10 vs. 1, *p* = .0001) and HSG (10 vs. 2, *p* = .0007). TVS detected more fibroids than HSC (17 vs. 5, *p* < .0001). Tubal obstruction was identified more often by HSG than HSC (19 vs. 5, *p* < .0001).

**Conclusions:**

TVS is superior for evaluation of myometrial pathology. HSG is superior for evaluation of tubal pathologies. Endometrial pathologies are best identified with HSC.

## Background

Infertility is defined as the inability for a couple to conceive a pregnancy following 1 year of unprotected vaginal intercourse [1]. It is estimated that 10–15 % of couples seek treatment for infertility [1–3]. It is generally considered appropriate to evaluate a couple for causes of infertility after 1 year of failed attempts at conception. However, given the inverse relationship of female fertility with age, it is often recommended that women over 35 years of age be evaluated after 6 months of failure to conceive, and women older than 40 be evaluated immediately [1].

A variety of factors may affect normal fertility including patient age, anatomy, ovulatory status, and sperm quality. Potential causes of infertility can be divided into male and female causes and include endocrine, anatomic, genetic, and behavioral conditions [4]. As a result, the evaluation of the infertile couple is multifactorial, necessitating physical examination, hormonal testing, and imaging. Because the infertility population is under a great deal of psychological and emotional stress, these patients benefit from a structured and streamlined evaluation. In particular, evaluation of the female partner attempting to conceive requires assessment of the uterus, endometrium, and fallopian tubes for anomalies or abnormalities potentially preventing normal conception. The best method for assessing these structures usually involves some combination of transvaginal sonography (TVS), hysterosalpingography (HSG), and hysteroscopy (HSC). Less often, pelvic magnetic resonance imaging (MRI) and saline infusion sonohysterography (SIS) are used.

The objective of this paper is to compare the diagnostic accuracy of TVS, HSG, and HSC for diagnosing uterine and tubal abnormalities in women with infertility to determine if all three modalities are necessary in the work up of these patients.

## Methods

We identified all baseline TVS performed on women in our infertility program from October 12, 2011 to October 12, 2013, prior to their initiation of assisted reproductive techniques (ART). From this group, we narrowed our patient population to those who had an HSG within 180 days of the TVS to maximize the likelihood of concordance between the studies. All TVS and HSG reports were reviewed for tubal, myometrial, or endometrial findings and anatomical variants.

We reviewed patient medical records to identify those patients who also had hysteroscopy (HSC) within 180 days of the baseline TVS, and we recorded the reported findings.

Myometrial abnormalities were categorized as fibroids or adenomyosis; endometrial abnormalities as polyps, cysts, cavity distortion (e.g., synechia, stricture), or nonspecific asymmetry; and tubal abnormalities as obstruction. For each abnormality, the frequency of detection by each modality (TVS, HSG, HSC) was tabulated. For TVS, visualization of a hydrosalpinx was classified as an obstructed fallopian tube. Detection rates of abnormalities were compared among the modalities using the Fisher exact test, with a *p*-value of <0.05 considered significant.

This study was approved by the Brigham and Women’s Hospital Institutional review board, protocol number 2014P000355.

## Results

A total of 1274 patients received a baseline TVS as part of a work up for infertility during the study period. Among these patients, 327 underwent a diagnostic HSG within a 180-day interval of the sonogram and comprise our study population. The time between TVS and HSG was 94 ± 49 days (mean ± SD). Among our 327 study patients, 55 also underwent HSC. The time (mean ± SD) between TVS and HSC was 61 ± 41 days and between HSG and HSC was 61 ± 50 days.

Among the study population of 327 patients (Table 1), 74 (23 %) had fibroids and 7 (2 %) had adenomyosis as diagnosed by either modality. Endometrial abnormalities were found in 16 (5 %) patients, based on TVS or HSG. Tubal obstruction was found in 56 (17 %) patients, more commonly unilateral (47 patients) than bilateral (9 patients).

**Table 1:** Myometrial, endometrial, and tubal abnormalities detected by transvaginal ultrasound and/or hysterosalpingography (*N* = 327)

Category	TVS	HSG	Statistical significance
Myometrium			
Fibroids	74 (23 %)	5	*p* < .0001
Adenomyosis	7 (2 %)	2	*p* < .0001
Cesarean scar	1	0	
Endometrium			
Polyps	4	4	
Cysts	4	0	
Cavity distortion	0	6	*p* < 0.002
Nonspecific asymmetry	0	2	
Tubes			
Total obstructed	8	56 (17 %)	*p* < .0001
Unilateral	7	47	
Bilateral	1	9	
Anomalies	4		

Abbreviations: *TVS* transvaginal ultrasound, *HSG* hysterosalpingography

**Fig. 1:**
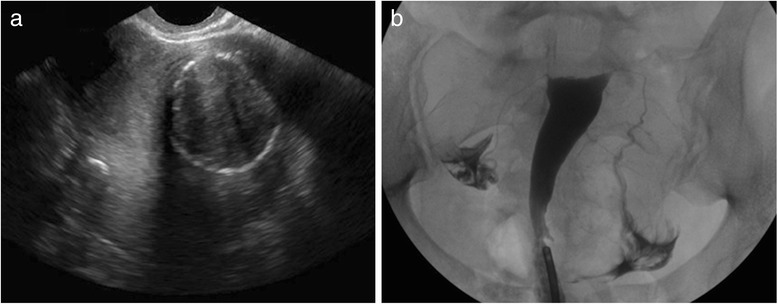
**a** 38-year-old G1P1 female with a history of infertility presenting for baseline assessment prior to initiation of ART. Coronal transvaginal sonographic image through the uterus demonstrates a 6.0 × 4.2 × 3.9 cm left sided mass with heterogenous echotexture and an echogenic rim, consistent with a large calcified intramural fibroid. **b** HSG demonstrates a normal endometrial cavity without filling defects to suggest fibroids as seen on TVS. The fallopian tubes are normal in caliber and demonstrate free intraperitoneal spill of contrast bilaterally

**Fig. 2:**
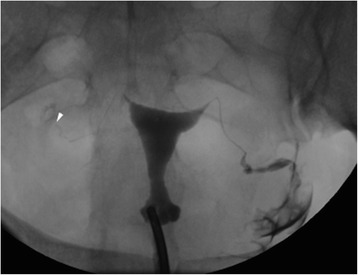
36 year-old G2P1A1 female with a history of infertility × 3 years, presenting for baseline assessment of tubal patency prior to initiation of ART. HSG demonstrates normal contour of the endometrial cavity. The left fallopian tube opacifies normally and demonstrates free intraperitoneal spill. The right fallopian tube fills with contrast, but terminates abruptly near its terminus (arrowhead). No right sided contrast spill is identified, diagnostic of distal tubal obstruction. This tubal obstruction was not appreciated on TVS or HSC

**Fig. 3:**
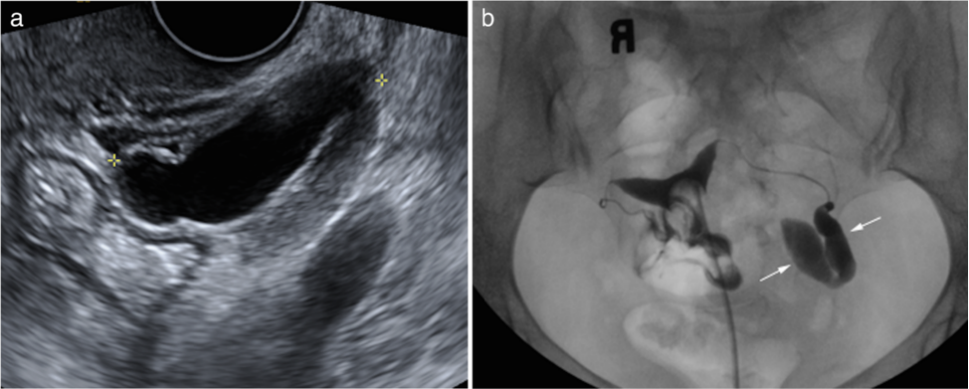
**a** 38-year-old G2P2 female with history of male factor infertility presenting for baseline assessment prior to initiating ART. Transvaginal grey-scale image of the left adnexa demonstrates an anechoic tubular structure (calipers), measuring 43 × 31 × 19 mm, separate from the left ovary (not shown) and consistent with hydrosalpinx. **b** HSG demonstrates contrast pooling within a dilated, blind ending fallopian tube (arrows), confirming the presence of a left sided hydrosalpinx. The right fallopian tube is normal in caliber and demonstrates free intraperitoneal spill of contrast, indicating tubal patency

TVS detected myometrial abnormalities significantly more often than did HSG, identifying fibroids in 74 patients while HSG only identified 5 (Fig. 1), and detecting adenomyosis in 7, while HSG detected only 2 (*p* < .0001 for both comparisons). Both HSG and TVS diagnosed endometrial polyps in the same 4 patients. HSG detected 6 patients with cavity distortion, while TVS found none of these (*p* < 0.002). With respect to tubal abnormalities, HSG performed significantly better than TVS, detecting tubal obstruction in all 56 (Fig. 2), while ultrasound only diagnosed 8 (*p* < .0001) (Fig. 3).

Among the subset of 55 patients who were evaluated by all three modalities, TVS, HSG, and HSC (Table 2), 17 (31 %) had fibroids and 6 (11 %) had adenomyosis. Endometrial abnormalities were found in 13 (24 %) patients and tubal obstruction in 19 (35 %).

**Table 2:** Myometrial, endometrial, and tubal abnormalities detected by each modality (*N* = 55)

Category	TVS	HSG	HSC	Statistical significance
Myometrium				
Fibroids	17 (31 %)	6	4	*p* < .0001 (TVS vs HSC)
Adenomyosis	6 (11 %)	0	0	
Endometrium				
Polyps	1	2	10	*p* = .0001 ( HSC vs TVS)
				*p* = .0007 (HSC vs HSG)
Cysts	1	0	0	
Cavity distortion	0	4	2	
Nonspecific asymmetry	0	2	1	
Tubes				
Total Obstructed	6	19 (35 %)	5	<0.0001 (HSG vs HSC)
Unilateral	6	16	5	
Bilateral	0	3	0	
Anomalies	0	1	31	

Abbreviations: *TVS* transvaginal ultrasound, *HSG* hysterosalpingography, *HSC* hysteroscopy

In this group, TVS detected myometrial abnormalities significantly more often than did HSC, which identified only 4 of the 17 patients with fibroids (*p* < .0001) and none of the patients with adenomyosis. With respect to endometrial abnormalities, HSC outperformed TSV and HSG, identifying 10 polyps, while TVS only detected 1 (*p* = .0001) and HSG only 2 (*p* = .0007). HSG outperformed HSC for tubal obstruction, which only detected 5 of the 19 patients with unilateral or bilateral obstruction (<0.0001)

## Discussion

Diagnostic imaging plays an important role in the assessment of women with infertility. Although no consensus protocol for work up of these patients exists, the majority of infertility patients undergo a baseline TVS and HSG. TVS is used for evaluating ovaries, fallopian tubes, and the adnexa and is a favored imaging modality in the infertility population because it is readily available, relatively low cost, and does not use ionizing radiation. TVS is the test of choice for diagnosing polycystic ovary syndrome [5], and is helpful for identifying endometriosis and the sequelae of PID. In addition, TVS is invaluable for monitoring ovarian folliculogenesis during treatment with ART [6–8]. In contrast, HSG provides information about tubal patency and uterine cavity abnormalities such as anomalies, polyps, synechiae, and adhesions, any of which could interfere with embryo implantation [9]. However, HSG offers limited evaluation of the cervix and myometrium and does carry the small risks of contrast reaction and of ionizing radiation exposure [10]. Besides TVS and HSG, supplemental evaluation with SIS and hysterosalpingo-contrast sonography (HyCoSy) is sometimes performed. These imaging procedures are becoming more popular because of their ability to combine TVS adnexal evaluation with HSG-like assessment of the uterine cavity, without the risks of contrast reactions and radiation exposure [11–13], but are not yet universally available.

MRI of the pelvis offers multi-planar imaging and does not require the use of ionizing radiation. It is an excellent modality for detecting endometriosis [5] and is helpful for determine the nature of uterine duplication anomalies, leiomyomas, and adenomysis [14–18]. MRI is also employed for evaluating intracranial causes of infertility, such as pituitary adenomas. However, due to its high cost and limited access, MRI is not typically used in the infertility assessment except for a specific indication requiring such imaging.

At our institution, we begin the infertility assessment with an HSG. If there is evidence of an abnormal uterine cavity from etiologies such as uterine septa, submucosal fibroids, synchiae, or polyps, HSC is then typically performed [19–21]. The standard practice at our institution is to perform HSC in the office setting, reserving operative HSC and laparoscopy for patients who are not able to tolerate office based procedures and for situations for which surgical correction is required, such as septoplasty for the correction of a subseptate uterus. Hysteroscopy is also preformed prior to ART if there is a 6 month or greater delay between the HSG and ART. TVS is obtained when patients begin ART, and continues during folliculogensis.

Our results indicate that TVS is superior to HSG for detection of myometrial pathology, including fibroids and adenomyosis. These results make intuitive sense, as TVS uses high frequency sound waves to evaluate the 3 dimensional volume and echotexture of the uterine tissue, while HSG uses radiographs and contrast dye to outline the endometrial cavity. By assessing the contour of the contrast-filled cavity, information about the surrounding myometrium can be inferred, but not diagnosed, because the tissue itself is not imaged directly. HSG may detect submucosal fibroids, but other myometrial pathology, such as intramural or subserosal fibroids, are likely to be missed. Similarly, TVS is superior to HSC, which visualizes the walls of the uterine cavity but cannot assess for lesions within the myometrium.

**Fig. 4:**
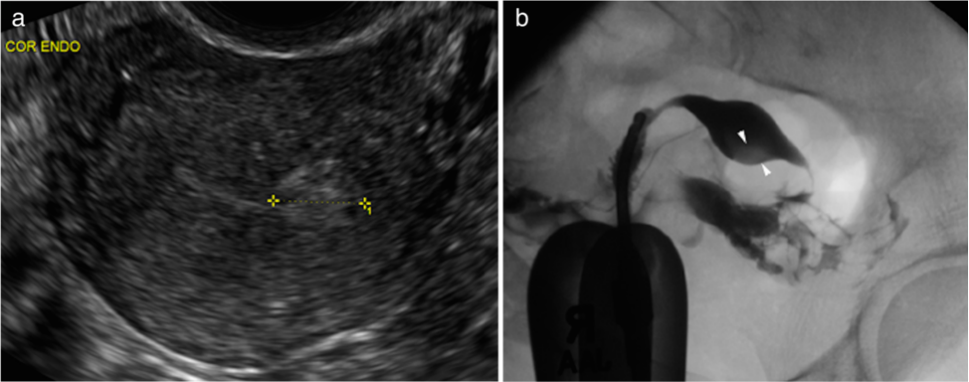
**a** 44 year-old G0P0 female with inability to conceive for 4 years presents for baseline assessment prior to IVF. TVS demonstrates an 11 × 11 × 10 mm echogenic lesion within the left aspect of the endometrial cavity (calipers). Flow was demonstrated within the lesion with color Doppler, raising the possibility of endometrial polyp. **b** Corresponding HSG demonstrates a depended rounded filling defect within the left aspect of the endometrial cavity, which persisted on multiple projections, suggestive of a polyp. The fallopian tubes are normal in caliber and patent. The patient went on to HSC, where the lesion proved to be a submucosal fibroid

Our results also indicate that HSG is the superior modality for detection of tubal pathology, specifically tubal obstruction. This finding is in keeping with the functional component of HSG, which allows the operator to visualize in real-time contrast medium passing through the tubes and most importantly, spilling into the surrounding peritoneum. TVS can only infer tubal obstruction when a hydrosalpinx is present, therefore obstructed but nondistended fallopian tubes will be missed with sonography alone. Endometrial pathologies, specifically endometrial polyps, were more frequently identified on direct visualization with HSC than on TVS and HSG combined. It is possible that, for some of our patients, the HSC preceded the TVS and/or HSG and, thus, polyps could have been removed by the time of imaging evaluation. While TVS and HSG are both potential screening modalities for endometrial lesions, HSC is required for optimal diagnosis (Fig. 4), and one reason why flexible office hysteroscopy remains the gold standard for endometrial assessment.

A weakness in our study is that we did not assess SIS as a method to evaluate the endometrium. This procedure is included in some protocols during the work up of women with infertility, but is not part of the routine assessment at our institution. SIS has been shown to be superior to TVS for identifying endometrial abnormalities including polyps and cavity distortion [11, 13, 22–27]. Some reports have also shown SIS to be comparable to the gold standard of HSC for evaulation of intrauterine abnormalities including polyps, submucosal fibroids, adhesions and uterine anomalies, with a sensitivity and specificity for detection of 88 and 94 %, respectively [28, 29]. In addition, none of our patients were evaluated by HyCoSy, a procedure that uses aerated saline or contrast to assess tubal patency with TVS. HyCoSy has been shown to be comparable to HSG with regards to assessing tubal patency, with sensitivity ranges from 75–96 % and specificity from 67–100 % [12, 13, 30, 31]. SIS and HyCoSy can be done in a single visit and together provide information about the uterine cavity and the patency of the fallopian tubes, similar to HSG, but with added information about the myometrium from the TVS component, all without exposure to ionizing radiation or iodinated contrast. Despite these advantages, HyCoSy does not provide anatomical information about the fallopian tubes, which limits its utility.

Given the lack of a single all encompassing imaging tool for accurately diagnosing endometrial, tubal, and myometrial causes of infertility, it could be helpful to outline one ste*p*-wise approach for use of the TVS, HSG, and HSC. Although there is tremendous variability between practices, at our institution most infertility patients undergo both a TVS and HSG prior to initiating ART. Others have found that SIS and HyCoSy provide comparable information as TVS and HSG combined. If findings of these tests suggest an abnormality within the uterine cavity, which could prevent implantation of a viable gestational sac, the patient will be referred for a HSC for direct inspection and possible treatment. However, the management of abnormal tubal pathology on HSG will vary depending on plan for reproductive therapy. If the patient is an In-vitro Fertilization (IVF) candidate, tubal obstruction is not of much consequence, as the embryo is directly implanted into the uterus. However, if the patient is not a candidate for IVF, tubal obstruction can be further managed with surgical interventions such as tuboplasty or salpingostomy.

## Conclusion

Our study compared the results from TVS, HSG, and HSC in a cohort of female infertility patients. TVS was superior for detecting myometrial pathology, HSG was superior for evaluating tubal patency, and HSC detected more endometrial polyps than HSG and TVS. No single modality provided accurate identification of all different pathologies. Complete work up of women with infertility may include all modalities, given the unique information obtained from each. However, with knowledge of the unique specificity of each imaging test to detect specific pathologies, a combination of HSG, HSC and TVS could be selected based on the clinical presentation of patients.
